# Reversible Block of Mouse Neural Stem Cell Differentiation in the Absence of Dicer and MicroRNAs

**DOI:** 10.1371/journal.pone.0013453

**Published:** 2010-10-18

**Authors:** Therese Andersson, Sabhi Rahman, Stephen N. Sansom, Jessica M. Alsiö, Masahiro Kaneda, James Smith, Donal O'Carroll, Alexander Tarakhovsky, Frederick J. Livesey

**Affiliations:** 1 Gurdon Institute, University of Cambridge, Cambridge, United Kingdom; 2 Department of Biochemistry, University of Cambridge, Cambridge, United Kingdom; 3 Laboratory of Lymphocyte Signalling, The Rockefeller University, New York, United States of America; University of Washington, United States of America

## Abstract

**Background:**

To investigate the functions of Dicer and microRNAs in neural stem (NS) cell self-renewal and neurogenesis, we established neural stem cell lines from the embryonic mouse Dicer-null cerebral cortex, producing neural stem cell lines that lacked all microRNAs.

**Principal Findings:**

Dicer-null NS cells underwent normal self-renewal and could be maintained *in vitro* indefinitely, but had subtly altered cell cycle kinetics and abnormal heterochromatin organisation. In the absence of all microRNAs, Dicer-null NS cells were incapable of generating either glial or neuronal progeny and exhibited a marked dependency on exogenous EGF for survival. Dicer-null NS cells assumed complex differences in mRNA and protein expression under self-renewing conditions, upregulating transcripts indicative of self-renewing NS cells and expressing genes characteristic of differentiating neurons and glia. Underlining the growth-factor dependency of Dicer-null NS cells, many regulators of apoptosis were enriched in expression in these cells. Dicer-null NS cells initiate some of the same gene expression changes as wild-type cells under astrocyte differentiating conditions, but also show aberrant expression of large sets of genes and ultimately fail to complete the differentiation programme. Acute replacement of Dicer restored their ability to differentiate to both neurons and glia.

**Conclusions:**

The block in differentiation due to loss of Dicer and microRNAs is reversible and the significantly altered phenotype of Dicer-null NS cells does not constitute a permanent transformation. We conclude that Dicer and microRNAs function in this system to maintain the neural stem cell phenotype and to facilitate the completion of differentiation.

## Introduction

Neural stem (NS) cells in the developing vertebrate embryo are multipotent, self-renewing cells that give rise to specific types of neurons in a fixed temporal order. MicroRNAs have been proposed as candidates for regulating many aspects of neural stem cell biology, based on the functions of microRNAs in the control of developmental timing and cell fate specification in a number of biological systems, including stem cells (for reviews, see [1], [2]). In the nervous system, microRNAs have been proposed to regulate stem cell self-renewal, neurogenesis, cell fate determination and neuronal differentiation [3], [4].

Many microRNAs are expressed in the developing forebrain, including microRNAs expressed in NS cells or during neuronal differentiation *in vivo* and *in vitro* [5], [6], [7], [8], [9]. Marked changes in microRNA expression occur during the induction of neurogenesis from embryonic stem (ES) cells and gain and loss of functions of single microRNAs can alter the degree of neurogenesis from ES cells [10]. Functional studies of neurogenesis in the olfactory epithelium have found that the major role of microRNAs and specifically the mir-200 family is to regulate neuronal differentiation in olfactory neurons, rather than regulate neurogenesis [11].

One approach to studying the possible functions of microRNAs has been to genetically ablate key proteins required for the biogenesis of microRNAs, most commonly the RNase III enzyme Dicer. Loss of Dicer function in embryonic stem (ES) cells revealed a requirement for Dicer and microRNAs in ES cell differentiation, but not ES cell maintenance and self-renewal [12], [13]. Null mutations in the Drosha-associated RNA binding protein DGCR8 gave a slightly different phenotype, in that ES cells failed to differentiate *in vitro* and also failed to down-regulate expression of key regulators of ES cell self-renewal [14]. Studies of loss of Dicer function in the olfactory epithelium [11] and the developing cerebral cortex [15], [16] have found that Dicer is not required for neurogenesis. Similarly, neurogenesis still takes place in the retina following Dicer ablation [17], [18].

However, *in vivo* studies of microRNA function by removal of Dicer share a common problem that deletion of Dicer does not immediately result in microRNA loss of function. MicroRNAs have been found to have relatively long half-lives in several different cell types and to persist for many cell cycles after Dicer loss of function [19]. In a developing neural stem cell-specific Dicer mutant, microRNAs persisted for several days after Dicer ablation [16], and we have also found that following Dicer deletion in many cases microRNAs can persist at over 40% of wild-type levels during the period of neurogenesis.

To study the absolute requirement for Dicer-dependent microRNAs and Dicer in neurogenesis and neural stem cell self-renewal, we generated NS cell lines lacking Dicer from the embryonic, Dicer-null cerebral cortex. It has recently been reported that Dicer-null NS cells can be generated from the Dicer-null cortex at low efficiency, and are impaired in their ability to self-renew and generate differentiated progeny [20]. We find that Dicer is not required for neural stem cell derivation. We cultured Dicer-null NS cells for several weeks to allow microRNA turnover to remove persisting microRNAs, generating Dicer-null NS cells that lack detectable microRNAs. We found that Dicer and microRNAs are dispensable for self-renewal, but that microRNAs are essential for the neural stem cell differentiation to either neurons or glia. This contrasts with previous *in vivo* findings [11], [15], but is consistent with studies of embryonic stem cells [12], [13], [14]. This difference is likely to be due to the ability to allow microRNAs to reduce to undetectable levels in Dicer-null NS cells in culture, whereas the long half-lives of microRNAs results in persistence of many microRNAs during the period of neurogenesis following Dicer deletion *in vivo.*

Dicer-null NS cells have an abnormal phenotype, combining increased expression of key regulators of stem cell maintenance with inappropriate expression of differentiation genes. Under neuronal differentiation conditions Dicer-null NS cells exhibit growth factor withdrawal-induced apoptosis. In three different glial differentiation conditions, Dicer-null NS cells do not undergo apoptosis, instead initiating but failing to complete the glial differentiation programme. Strikingly, the ability of Dicer-null NS cells to generate both neurons and glia is restored by the acute reintroduction of Dicer, demonstrating that these cells are not irreversibly transformed despite the absence of Dicer and microRNAs. We conclude that removal of Dicer and microRNAs substantially alters the phenotype of NS cells to render them incapable of full differentiation, but that the ability to differentiate is restored by the acute reintroduction of Dicer, indicating that the altered phenotype of Dicer-null NS cells is not a permanent transformation.

## Materials and Methods

### Generation of cortex-specific Dicer-null mice

Nestin-Cre transgenic mice [21] were crossed to Dicer^fl/fl^ mice [22] to produce Nes-Cre; Dicer^fl/+^ founder males. Nes-Cre; Dicer^fl/+^ males were crossed with Dicer^fl/fl^ female mice to generate Dicer-null embryos. All procedures were carried out according to UK Home Office regulations, under UK Home Office Project Licence PPL/182193, and approved by the Gurdon Institute animal use committee. Mice were genotyped for the Nes-Cre transgene and the Dicer alleles by PCR [21], [22].

### Neural stem cell line derivation, culture and characterization

Polyclonal neural stem cell lines were established from the E12.5 cortex of Dicer-null and wild-type littermate mice and maintained according to published methods with minor variations [23]. Briefly, E12.5 cortices were dissociated to single cells and placed in non-adherent culture conditions to promote neurosphere formation in the presence of 10 µg/ml recombinant EGF and 10 µg/ml recombinant FGF2 (Peprotech). Neurospheres were harvested seven days later and transferred to laminin-coated culture dishes to promote adherence and cellular outgrowth. Karyotyping was carried out by DAPI staining of colcemid-treated cells, with at least five clear chromosome spreads counted for each line analysed. Neuronal differentiation of NS cells was initiated by sequential withdrawal of EGF and FGF2, essentially as described [23]. Glial differentiation was induced either by FGF2 withdrawal or addition of either 2% foetal calf serum (FCS; Sigma-Aldrich) or 10 µg/ml BMP4 (Peprotech) to the culture medium.

For immunofluorescent staining, cultures were fixed with 4% paraformaldehyde in PBS or in ice-cold methanol. Primary antibodies used in this study were: Nestin (mouse; BD Biosciences), Sox2 (goat; Santa Cruz Biotechnology), Tuj1 (mouse; Covance), H3K9Me3 (rabbit; Upstate), GFAP (rabbit; Sigma-Aldrich), Hmga2 (rabbit; kind gift of Dr. M. Narita), Cyclin D1 (mouse; Santa Cruz Biotechnology). Primary antibodies were detected with species-specific Alexa-488 or Alexa-546 conjugated secondary antibodies. Immunolabelled cells were visualised by confocal or epifluorescence microscopy.

### Microarray analysis of microRNA and mRNA expression

Custom microarrays representing 171 microRNAs in the microRNA registry, 3 snRNAs and 4 tRNAs were constructed using 5′-amine-linked antisense oligonucleotides printed on CodeLink slides. Short RNAs were extracted from embryonic cortex and cells in culture (mirVana, Ambion), visualised on a BioAnalyzer (Agilent) and quantified with a Nanodrop spectrophotometer. 250 ng of short RNAs were labelled (Array 900RT, Genisphere) and hybridised overnight before detection according to the manufacturer's instructions. For messenger RNA expression profiling, total RNA isolated from neural stem cell cultures was used to generate cDNA by reverse transcription and PCR amplification using the SMART system (BD) [24]. Amplified cDNA was labelled by the incorporation of either Alexa 555 or 647-dCTP (Invitrogen), as previously described [25], [26]. The microarrays used for this study were arrays of approximately 22,500 65-mer oligonucleotides (Compugen/Sigma-Genosys) printed in-house on CodeLink slides (Amersham) or arrays of the MEEBO oligonucleotide library (Invitrogen). Array slides were scanned in an Axon GenePix 4000B scanner (Axon Instruments), and data from each array extracted using Genepix 4.0 (Axon Instruments). Microarray data were archived and analysed locally in Acuity (Axon Instruments) and in Bioconductor.

### Microarray data analysis and bioinformatics

MicroRNA levels in Dicer-null NS cells were normalised to those in wild-type NS cells using the set of control small RNAs on the array (tRNAs, snRNAs). MicroRNA levels were expressed relative to wild-type levels, correcting for local and systemic background signals. Gene expression differences between wild-type and Dicer-null self-renewing NS cells were identified by comparing two wild type and two Dicer-null NSC lines on six dye-swapped MEEBO microarrays: four hybridisations directly compared expression between Dicer-null and wild-type lines, and two hybridisations directly compared the two wild-type lines. Expression analysis was carried out using the Limma package[27]. Low intensity features were identified and weighted appropriately, arrays were lowess normalised and then normalised between arrays. Control features and features not representing genes were excluded before the identification of differentially expressed genes using the eBayes algorithm. An adjusted p-value cut-off of 0.05 was used to identify significant genes. Gene ontology analysis of the gene expression changes in Dicer-null NS cells was performed using GOToolBox (http://crfb.univ-mrs.fr/GOToolBox/home.php) [28]. GO categories (p<0.01) significantly enriched by comparison with the whole probeset were identified using a Benjamini & Hochberg corrected hypergeometric test.

To compare gene expression changes between wild-type and Dicer-null NS cells during glial differentiation, RNA was harvested from wild-type and Dicer-null NS lines 24 hours after the addition of BMP4 or PBS. A set of six array hybridisations was carried out: two hybridisations comparing gene expression between wild-type NS cells with and without BMP4 treatment; two hybridisations comparing gene expression between Dicer-null NS cells with and without BMP4 treatment; and two hybridisations directly comparing BMP-treated wild-type and Dicer-null NS cells. Array data were lowess normalised (in Acuity) and the entire set of six arrays analysed as a group. Data were filtered to remove low-intensity features and features absent on more than two arrays. To identify robust changes in gene expression occurring in at least one of the cell lines, only genes showing at least a 50% change in gene expression in two or more arrays were retained for further analysis. The content of this final set of genes was explored by hierarchical and agglomerative clustering in the Acuity system to identify the major classes of gene expression changes, as reported above. To prospectively identify differences in the response to BMP4 treatment between wild-type and Dicer-null NS cells, we used the significance analysis of microarrays (SAM) algorithm [29] to analyse the four microarrays comparing BMP-treated and control Dicer-null and wild-type NS cells. Data were normalised and filtered as before and significant changes in gene expression discovered by performing a two-class SAM, using a false discovery rate (FDR) cut-off of 0.1. Data passing the 0.1 FDR were hierarchically clustered using MEV to aid visualisation [30].

### Retroviral expression of Dicer

Human full-length Dicer (hDicer, kindly provided by Dr P. Provost, Laval) was subcloned into the MMLV-based pFB murine replication-incompetent retrovirus plasmid vector (Stratagene). The pFB-hDicer construct was introduced into 293T cells together with the pCL-Eco packaging plasmid by calcium phosphate transfection. Virus-containing supernatants were collected 36 and 48 hours after transfection, centrifuged and directly added to NS cultures. Cells were washed and the culture media replaced 24 or 48 hours after application of virus to initiate neuronal or glial differentiation. Viral infection and Dicer expression in infected cells was confirmed by genomic PCR and RT-PCR for hDicer using two different sets of primers specific to human Dicer.

## Results

### Neural stem cell lines can be established from the Dicer-null brain and self-renew normally

**Figure 1:**
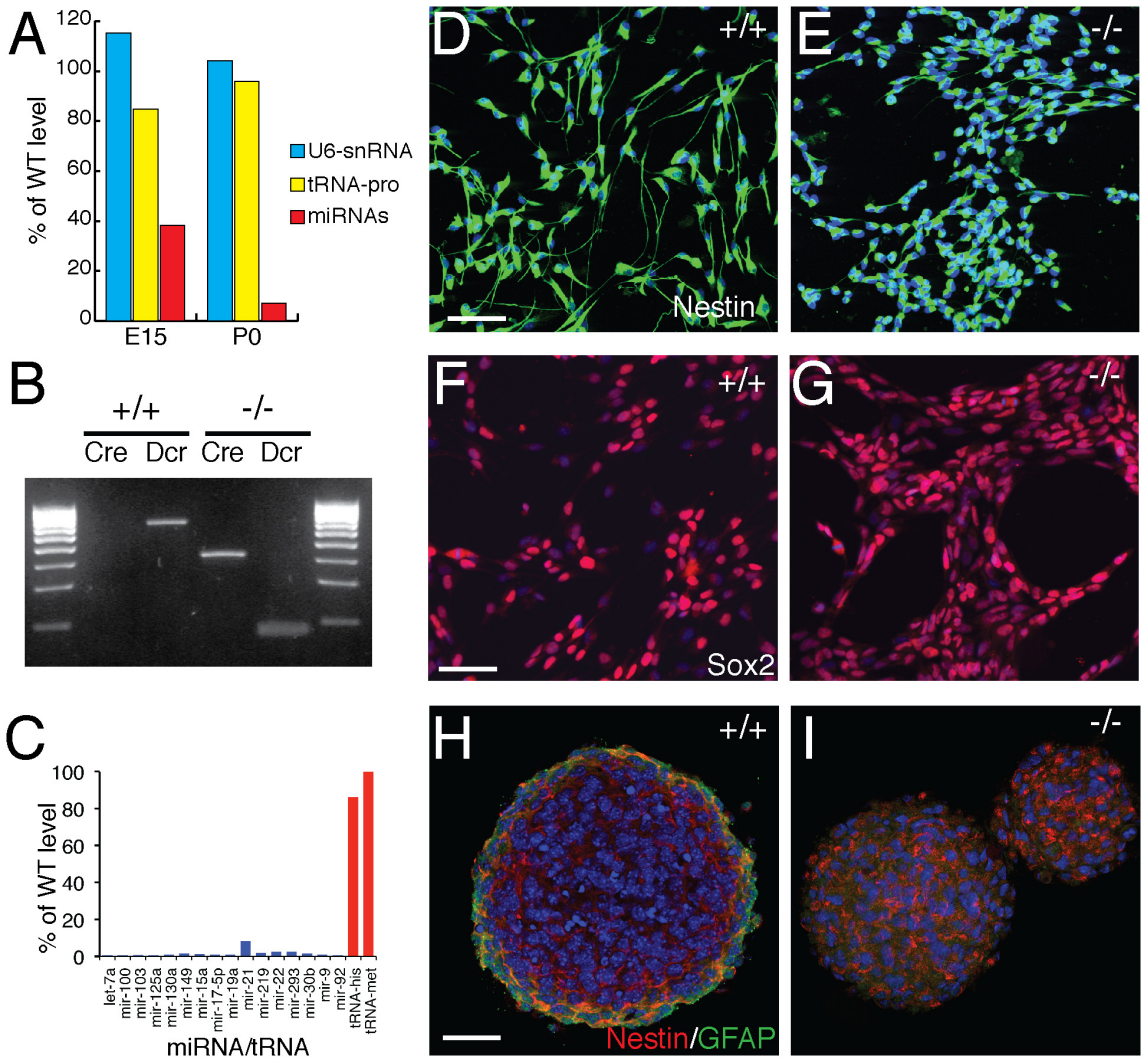
Dicer-null cortical neural stem cells can be established from the embryonic cerebral cortex and self-renewal appropriately. (A) At E15, several days after Dicer ablation using the neural progenitor Nes-Cre deleter line, Dicer-null cortices still have about 40% of wild-type microRNA levels (average of the relative expression of all microarray–detected microRNAs), whereas microRNAs are almost undetectable in the cortex at birth. MicroRNA levels were measured as described in the methods and Table S1. (B) PCR genotyping of monolayer cultures of cortex-derived NS cell lines demonstrates that lines derived from Nes-Cre;Dicer^fl/fl^ E12 cortex are Cre+ and contain only the recombined Dicer allele. (C) Dicer-null NS cells lack expression of microRNAs, compared to wild-type cells, although expression of other short RNAs, such as tRNAs, is normal. Expression for a subset of typical microRNAs and two tRNAs is shown relative to expression in control NS cells, and is the average of six replicate features on the array. (D–G) Neural stem cell lines established from wild-type and Dicer-null E12.5 cortex in monolayer culture both express the NS-cell specific intermediate filament protein Nestin (D, E) and the NS cell transcription factor Sox2 (F, G). While Nestin is clearly expressed, the morphology of Dicer-null NS cells is abnormal and lacks the long radial fibres found in wild-type NS cells. Scale bars, 25 µm. (H, I) Dicer-null and control NS cells both form neurospheres in non-adherent culture, in which the majority of cells express Nestin. Note that cells at the periphery of neurospheres generated by control cells begin to differentiate to astrocytes, as indicated by GFAP expression, whereas no GFAP expression is observed in Dicer-null neurospheres. Scale bar, 25 µm. Nuclei in all images visualised with DAPI.

Microarray analysis of microRNA expression at late stages of neurogenesis in the cerebral cortex of a nervous system-specific Dicer mutant (Nes-Cre/Dicer; embryonic day 15, E15) found that microRNAs were still present in the Dicer mutant cortex at a significant level, typically around 35–40% of wild-type littermate levels (Fig. 1A). In contrast, microRNAs were effectively undetectable in the Dicer-null cortex at birth (Fig. 1A). Therefore, it was not possible to analyse the effects of the complete absence of microRNAs on neural stem cell self-renewal and neurogenesis during development *in vivo*. To investigate whether microRNAs are required for neural stem and progenitor cell self-renewal, proliferation and neurogenesis, we derived neural stem (NS) cell lines from the cerebral cortex of Dicer mutants and control littermates at E12.

NS cell lines were established from Dicer-null cortices at the same efficiency as homozygous floxed Dicer allele control littermates (Fig. 1) and confirmed as being Dicer-null by genotyping (Fig. 1B). Cells of both genotypes were maintained for several weeks and the absence of microRNAs from the Dicer-null cells confirmed by array analysis (Fig. 1C; Table S1). Characterisation of the effects of Dicer and microRNA loss on neural stem cell biology focussed on two independent Dicer-null NS lines and two independent homozygous floxed Dicer allele control lines. Both control and Dicer-null NS lines expressed proteins characteristic of NS cells, including the intermediate filament protein Nestin and the transcription factor Sox2 (Fig. 1D–G). Dicer-null NS cells were morphologically different to control cells, lacking the long, fine radial processes seen in control cells, instead having shorter, twisted processes (Fig. 1D, E).

The ability of control and Dicer-null NS cells to form primary and secondary neurospheres was confirmed for both low and high passage number cells (Fig. 1H, I). Therefore, we conclude that NS cell lines can be established from the embryonic Dicer-null cerebral cortex, and that those NS cells can self-renew appropriately, despite the lack of Dicer and all microRNAs.

### Dicer-null NS cells show subtly altered cell cycle kinetics and heterochromatin organization

**Figure 2:**
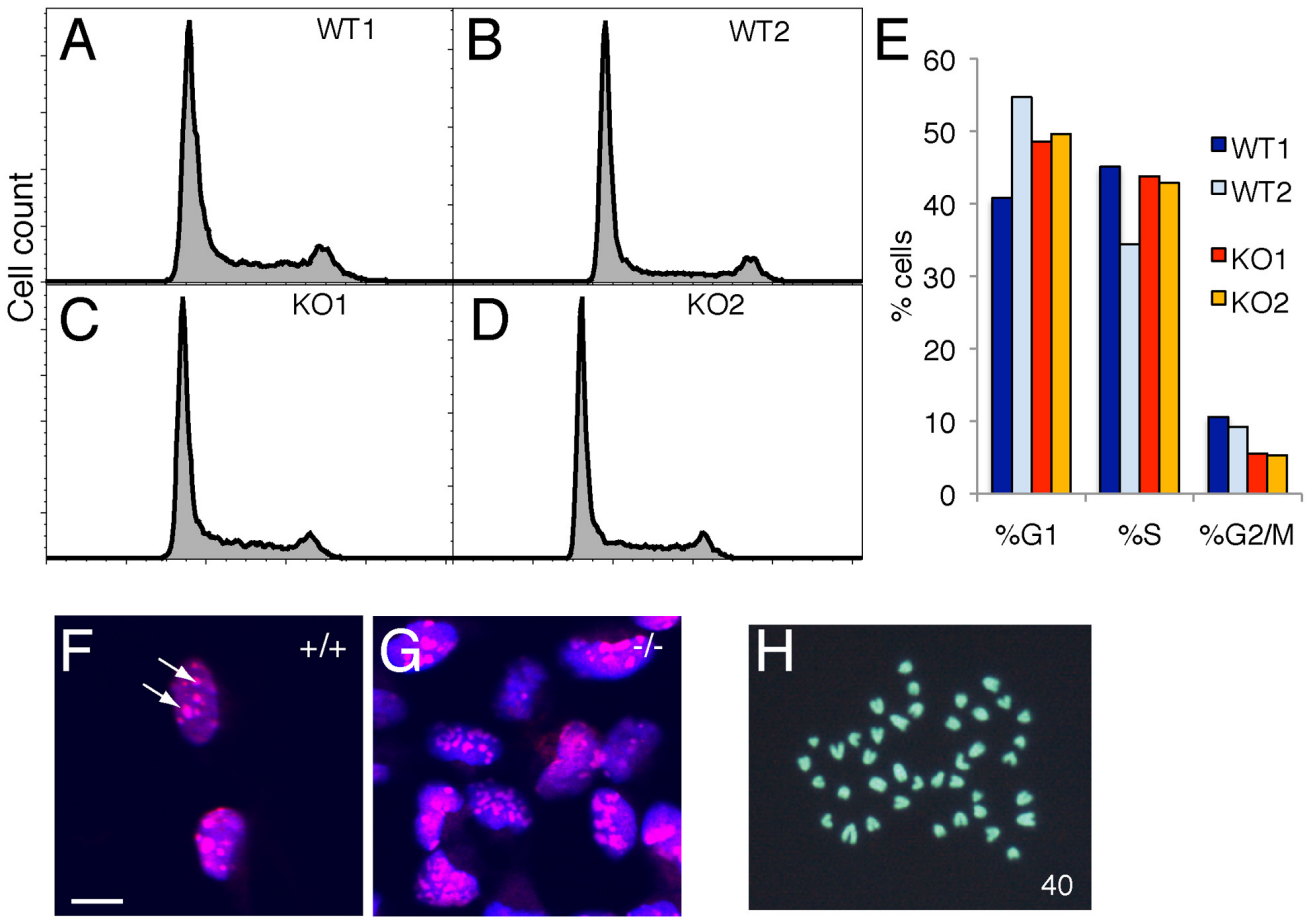
Dicer-null NS cells have minor alterations cell cycle kinetics and significant changes in heterochromatin organisation. (A–D) Plots of the cell cycle distributions of wildtype (A, B) and Dicer-null (C, D) cells. In both genotypes the majority (∼60%) of cells are in G1, reflecting the relatively slow cycle times for NS cells. Y-axis, cell counts; x-axis, DNA content (Hoechst 33342 fluorescence). (E) Histogram of the proportions of NS cells in each phase of the cell cycle. There is a significant difference in the numbers of cells in G1 in the two control lines, however there is a consistent decrease in the proportion of Dicer-null NS cells in G2/M, compared with controls. (F, G) High-power, confocal images of wild-type (F) and Dicer-null (G) NS cells stained for H3K9Me3. Representative images are shown of the distribution of H3K9Me3 staining in control cells as a relatively small number of large foci (F, arrows). In contrast, the H3K9Me3 distribution is variable in the Dicer-null NS cells, with the majority of cells containing smaller, irregular foci of H3K9Me3 (G). Scale bar, 10 µm. (H) Dicer-null NS cells are karotypically normal, containing the normal complement of 40 chromosomes for mouse and no obvious translocations.

ES cells lacking microRNAs due to deletion of the DGCR8 gene, essential for microRNA biogenesis, have been reported to have changes in cell cycle kinetics, with an increase in the number of cells in G1 phase of the cell cycle [14], [31]. Analysis of the cell cycle distribution of two Dicer-null NS cells found that this was broadly similar to that observed in two control, wild-type NS lines (Fig. 2A–D). However, it is noteworthy that the two wild-type lines show some variation in their cell cycle kinetics (Fig 2A, B). In both null lines, reproducible but subtle changes in cell cycle distributions were observed, most notably a reduction in the proportion of cells in G2/M (Fig. 2E), suggesting that these cells are cycling slightly more slowly than controls. This is consistent with the longer time we observed for Dicer null NS cells to reach confluency in culture.

Dicer has been found to be important for the maintenance of heterochromatin in several vertebrate cell types, including ES cells [13], [32], [33]. Immunofluorescence analysis of H3K9Me3 levels and localisation in Dicer-null NS cells found that, although total H3K9Me3 levels were broadly similar to those seen in control NS cells, as previously observed [20], the distribution of nuclear H3K9Me3 was highly abnormal. Instead of a small number of relatively large H3K9Me3 foci, Dicer-null NS nuclei typically contain over twice as many H3K9Me3 foci and these are small and irregular in morphology (Fig. 2F, G), suggesting that Dicer-null NS cells fail to maintain heterochromatin appropriately. However, despite this, karyotype analysis demonstrated that Dicer-null NS cells had the normal complement of 40 chromosomes for mouse with no obvious translocations (Fig. 2H).

### Dicer-null NS cells fail to generate differentiated progeny

**Figure 3:**
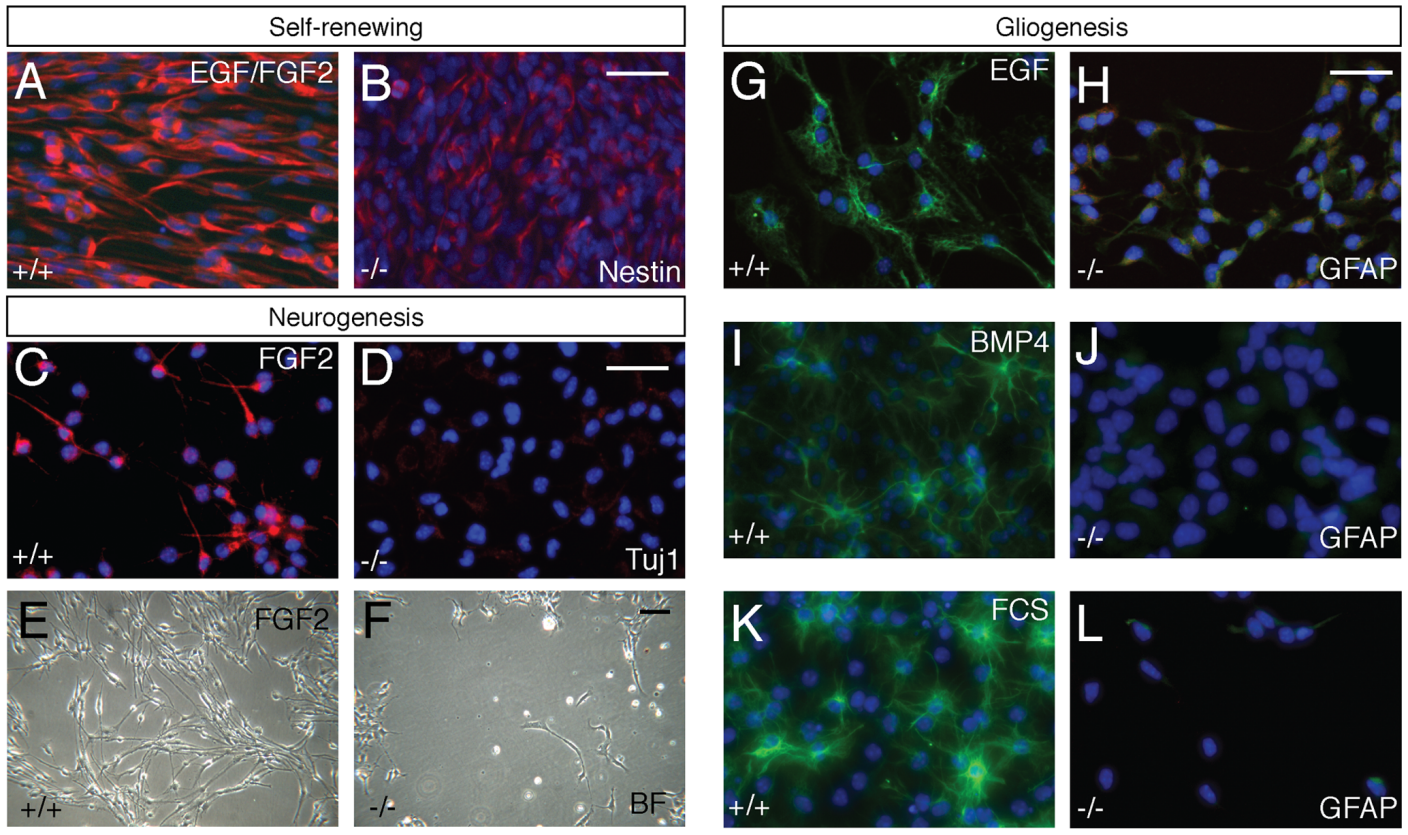
Dicer-null NS cells are incapable of neurogenesis and gliogenesis. (A, B) Under self-renewing conditions, Dicer-null NS cells express Nestin, but at a lower level than control NS cells. (C–F) Withdrawal of EGF and continued exposure to FGF2 promotes neurogenesis in control NS cells (Tuj1+ cells, C), but does not result in neurogenesis in Dicer-null NS cells (D). Shortly after EGF withdrawal most Dicer-null NS cells die and detach from the culture plate. Brightfield image of wild-type (E) and Dicer-null (F) 24 hours after EGF-withdrawal. (G, H) Withdrawal of FGF2 and continued exposure to EGF promotes gliogenesis in control NS cells (GFAP+ cells, G), but does not result in gliogenesis in Dicer-null NS cells (H), based on both GFAP expression and cell morphology. (I, J) Control NS cells generate large numbers of GFAP+ astrocytes 48 hours after exposure to BMP4 (I), whereas Dicer-null NS cells fail to express GFAP or assume an astrocytic morphology (J). (K, L) Addition of foetal calf serum (FCS) promotes gliogenesis from control cells within 48 hours (K), but does not stimulate gliogenesis from Dicer-null NS cells (L). Scale bar for all images, 25 µm. Nuclei in all images visualised with DAPI.

To test the ability of Dicer-null NS cells to generate differentiated progeny, we placed NS cells in a range of different conditions that drive gliogenesis or neurogenesis, using approaches that depend either on growth factor withdrawal or on positive, instructive signals. Neurogenesis from NS cell cultures can be induced by sequential withdrawal of two growth factors, EGF and FGF2, from the culture medium [23]. Using this approach, cells with classic neuronal morphology, expressing neuron-specific tubulin were observed within approximately six days in control cultures (Fig. 3E). The same procedure resulted in apoptosis of Dicer-null NS cells upon withdrawal of the first growth factor, EGF, such that there were very few live cells in the culture within 2–3 days and the few surviving cells did not differentiate into neurons (Fig. 3D, F).

We used three different methods to induce gliogenesis from NS cells: positive instruction by BMP4 or foetal calf serum (FCS), and selective growth factor withdrawal (removal of FGF2 and maintenance of EGF). In control cultures almost every cell had the classic astrocytic morphology and was strongly positive for the astrocyte intermediate filament protein GFAP within 48 hours of BMP4 or FCS addition to the cultures, or 96 hours of FGF2 withdrawal (Fig. 3G, I, K). In contrast, none of the Dicer-null NS cells expressed GFAP upon addition of BMP4 (Fig. 3J). The use of alternative methods for inducing gliogenesis, by withdrawal of FGF2 or the addition of foetal calf serum, completely failed to induce gliogenesis in one line of Dicer-null NS cells and did so at very low efficiency in the other (Fig. 3H, L and data not shown). It is noteworthy that we did not observe large-scale apoptosis under these differentiation conditions, and particularly upon FGF2 withdrawal, in contrast with the apoptosis observed upon EGF withdrawal.

### Dicer-null NS cells exhibit complex changes in mRNA levels resulting in a substantially altered phenotype

Given the functions of miRNAs in post-transcriptional gene silencing, loss of Dicer and all microRNAs would be expected to result in considerable alterations in mRNA levels and associated phenotypic changes. To assess whether this is the case, mRNA levels in two independent Dicer-null NS lines were compared with matched control NS lines. To control for differences in mRNA levels that were not due to the absence of Dicer and microRNAs, the analysis included a direct comparison of gene expression between the two control lines.

**Figure 4:**
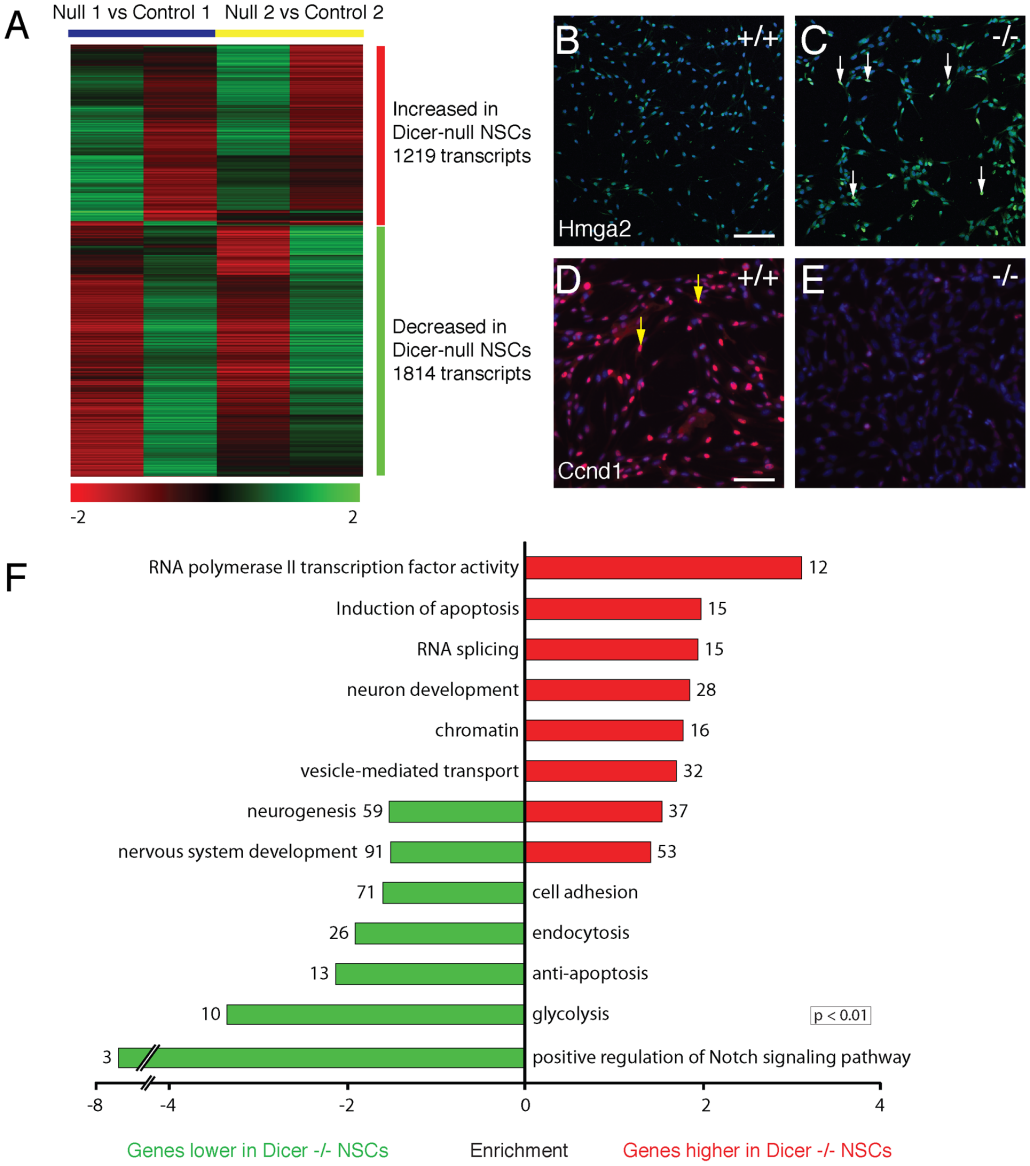
Dicer-null NS cells exhibit complex changes in mRNA levels resulting in a substantially altered phenotype. (A) Dicer-null NS cells show significant changes in the mRNA levels of 3033 genes, compared with control NS cells. Although a small number of transcripts demonstrated over ten-fold alterations in expression, the overwhelming majority showed changes of two-fold or less. See methods for technical details of the array hybridisations and statistical analysis. Detailed gene lists are provided in Table S2. (B–E) Changes in mRNA transcripts were reflected in changes in protein expression in cultures of Dicer-null NS cells. Increased Hmga2 mRNA was found to result in the appearance of a subset of strongly HMGA2-expressing nuclei in Dicer-null NS cells, as indicated by the arrows (B, C). Reduced levels of Cyclin D1 mRNA was reflected in the absence of CyclinD1 protein-positive Dicer-null NS cells, compared with controls (yellow arrows in D, with no Ccnd1+ cells detected in the Dicer-null cells in E). Antibody staining is representative of that seen in at least two cultures in two independent lines for each genotype. Scale bar, 25 µm. Nuclei are visualised with DAPI counterstaining. (F) Key Gene Ontology categories found enriched in the sets of genes up (red) and down-regulated in Dicer-null NS cells, compared to wild-type controls. For each category, the number represents the number of genes identified in that category, and the bar size reflects the fold-enrichment of that category in the up- or down-regulated gene set compared with the whole probeset. Details of all enriched GO categories are provided in Table S3.

Array analysis of mRNA expression in Dicer-null NS cells revealed significantly altered expression of 3033 transcripts, 1219 of which were increased and 1814 of which decreased in levels in Dicer-null NS cells (Fig. 4A; Table S2). Notable among the transcripts increased in levels in Dicer-null NS cells were stem cell-related transcripts, such as Prominin-1 (CD133) and Hmga2 (Fig. 4B, C), and a number of positive and negative cell cycle regulators, including cyclins E1 and B2 and Cdkn1c. However, there was also an increase in expression of genes normally specifically expressed in differentiating cortical neurons, including Foxp1, Foxp2, Doublecortin, Mtap1b and Enc1 [34], [35]. This was accompanied by down-regulation of other cell cycle regulators, including Cdkn1a and CyclinD1 (Fig. 4D, E and Table S2). Together, these indicate that the overall phenotypic change in Dicer-null NS cells does not correspond to a simple shift towards or away from a more differentiated phenotype.

To investigate in an unbiased manner whether there were clear trends in the cellular processes altered in Dicer-null NS cells, we carried out a Gene Ontology analysis of the sets of increased and decreased transcripts. Gene Ontology analysis found significant enrichments for sets of genes involved in several biological processes, suggesting several possible changes in the phenotype of Dicer-null NS cells (Fig. 4F; full details of the GO analysis are included in Table S3). Positive regulators of apoptosis are enriched in genes upregulated in Dicer-null NS cells, whereas negative regulators of apoptosis are enriched in the genes down-regulated in Dicer-null NS cells (Fig. 4F). This is consistent with the extreme sensitivity these cells display to EGF withdrawal. Underlining the complex nature of the phenotype of Dicer-null NS cells, genes involved in neurogenesis and nervous system development are enriched in both the up- and down-regulated gene sets, as are cell cycle regulators and regulators of the cytoskeleton.

### Dicer-null NS cells initiate but fail to complete astrocyte differentiation programs

**Figure 5:**
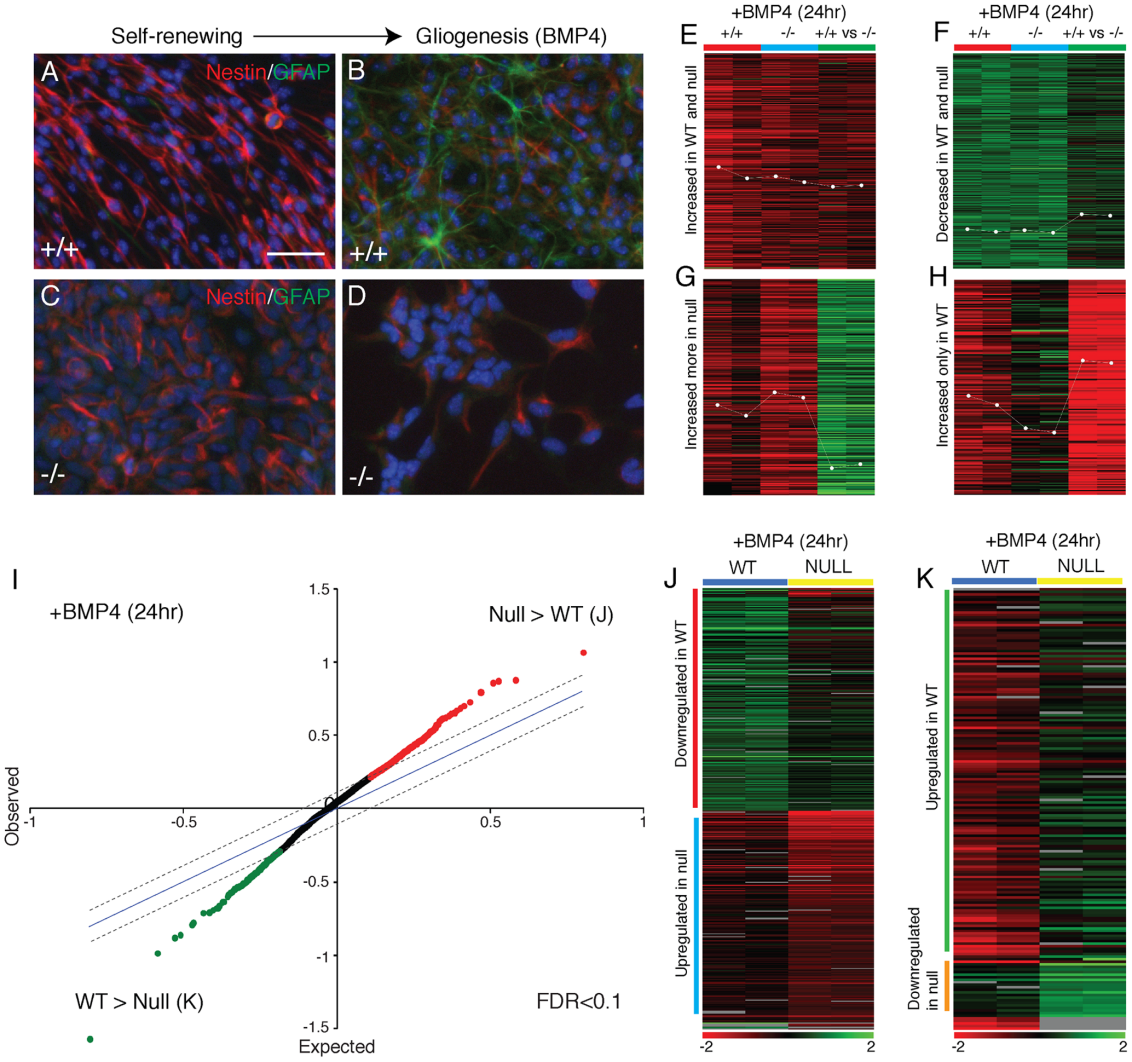
Dicer-null NS cells initiate but fail to complete a differentiation program. (A–D) Following two days of astrocytic differentiation in response to BMP4, Nestin protein is down-regulated in both control and Dicer-null NS cells, whereas the expression of the astrocyte-specific protein GFAP is only induced in control NS cells. Scale bar, 25 µm. (E–H) Microarray analysis of BMP4-induced glial differentiation in control and Dicer-null NS cells. Examples of the four major patterns of gene expression observed within 24 hours of BMP4 exposure are shown. In each cluster, each column represents a microarray experiment, each row a unique gene. Two hybridisations for each comparison are shown: control cells 24 hours after BMP4 exposure compared with untreated control cells (+/+); Dicer-null cells 24 hours after BMP4 exposure compared with untreated Dicer-null cells (−/−); BMP4-treated wild-type compared with BMP4-treated Dicer-null cells (+/+ vs −/−). Clusters of transcripts upregulated in both cell types (E) and down-regulated in both cell types (F) are shown, demonstrating that Dicer-null NS cells do execute components of the astrocytic differentiation program. However, as shown in (G) Dicer-null cells also upregulate a set of genes to an inappropriately high level, and also fail to upregulate a large set of genes that are upregulated in control cells (H). (I) Significance analysis of microarrays (SAM) plot comparing gene expression between BMP4-treated wild-type and Dicer-null NS cells (see methods for details). At a 0.1 false discovery rate, two sets of transcripts showing differential expression between the two groups of arrays (BMP4-treated Dicer-null/untreated Dicer-null vs BMP4-treated wild-type/untreated wild-type) were observed: genes more highly expressed in BMP-treated Dicer-null cells compared with the equivalent wild-type cells, and genes more highly expressed in BMP4-treated wild-type cells compared with the equivalent Dicer-null cells. (J, K) Hierarchical clustering of the two sets of transcripts detected by the SAM analysis reported in (I). Within each set, two subclusters were found and are labeled: transcripts higher in BMP4-treated Dicer-null cells contained a cluster of genes down-regulated only in the wild-type cells, but not in the Dicer-nulls (J), and a cluster of genes up-regulated in the Dicer-null cells, but not in the wild-type cells (J); similarly, transcripts higher in BMP4-treated wild-type cells contained a cluster of genes up-regulated only in the wild-type cells, but not in the Dicer-nulls (K), and a small cluster of genes down-regulated in the Dicer-null cells, but not in the wild-type cells (K). Cluster contents are detailed in Table S4.

Dicer-null NS cells completely fail to undergo neurogenesis and are incapable of gliogenesis. However, Dicer-null NS cells do change in morphology and express GFAP in a very small number of cells when placed in glial differentiation promoting conditions, suggesting that they do respond to differentiation signals and can, at very low frequency, complete this differentiation program. To address this, we studied the global gene expression changes that take place in control and Dicer-null NS cells when induced to differentiate. To do so, we used BMP4-induced astrocytic differentiation as a model, as this is an efficient system, with almost 100% of wild-type cells differentiating to astrocytes over 48 hours. Furthermore, this differentiation procedure does not involve EGF withdrawal, which causes massive apoptosis in Dicer-null NS cells (see above). Within 24 hours of BMP4 addition, Dicer-null NS cells down-regulate expression of the diagnostic neural stem cell gene Nestin, (Fig. 5A–D), indicating that the Dicer-null NS cells can respond to differentiation signals appropriately.

To explore the progression of the BMP4-induced astrocyte differentiation program in wild-type and Dicer-null NS cells, we applied microarray gene expression profiling to study gene expression in the first 24 hours following BMP4 treatment (Fig. 5E–H). Gene expression in BMP4-treated Dicer-null and wild-type NS cells was compared to their untreated counterparts and the expression data first explored by clustering methods to identify the broad patterns of gene expression changes following BMP4 treatment. The array analysis found that Dicer-null NS cells increase expression of approximately half of the genes that are seen to upregulate in control cells and also down-regulate many of the genes that control cells down-regulate upon differentiation (Fig. 5E, F). However, Dicer-null NS cells fail to activate expression of a large set of genes expressed during glial differentiation, including GFAP (Fig. 5H). Furthermore, there is a set of genes inappropriately increased in expression in Dicer-null NS cells that are not induced in wild-type NS cells (Fig. 5G).

To directly identify the differences in the transcriptional responses of Dicer-null and wild-type NS cells to BMP4, a statistical analysis using the significance analysis of microarrays algorithm (SAM, see methods for details) was carried out (Fig. 5I). A set of 616 transcripts showing significant (false discovery rate <0.1) differences between the BMP4-treated Dicer-null and wild-type NS cells was hierarchically clustered to aid visualisation of the different classes of expression differences detected (Fig. 5J, K). Four classes were detected: transcripts down-regulated only in the wildtype cells after 24 hours of BMP4 exposure, with no change in expression in Dicer-null NS cells (227 transcripts); transcripts up-regulated only in the Dicer-null cells after 24 hours of BMP4 exposure, with no or significantly lower change in expression in wild-type NS cells (218 transcripts); transcripts only upregulated in wild-type cells (150 transcripts); transcripts downregulated only in Dicer-null cells (21 transcripts). Details of the contents of the SAM clusters (Fig. 5J, K) are given in the supplementary material (Table S4).

The set of genes showing differential expression between Dicer-null and wild-type NS cells was compared to published data on genes specifically expressed in differentiating astrocytes [36]. Of the top 40 genes reported as having astrocyte-specific expression, 8 (20%) are found in the set of genes upregulated in wild-type cells and not by Dicer-null cells (Table S5), including GFAP, Aqp4, Slc1a2 and AldoC [36]. None of the astrocyte-specific genes were found in the other clusters. Therefore, we conclude that Dicer-null NS cells do respond to differentiation signals appropriately and initiate a differentiation program. However, they fail to complete this programme and do not reach the terminally differentiated phenotype.

### The Dicer-dependent block in NS cell differentiation is reversed by the reintroduction of Dicer

The phenotype of Dicer-null NS cells reported here is complex in its nature: these cells have an abnormal morphology, disrupted heterochromatin, markedly different gene and protein expression, a dependency on EGF for survival and an inability to differentiate. The gene expression phenotype of Dicer-null NS cells alone indicates that these cells are highly abnormal. Therefore, it is possible that the inability to differentiate reflects a pathological, irreversible transformation of these cells. Of particular concern in this respect is the significant upregulation in Hmga2 mRNA and protein levels, given the oncogenic role of Hmga2 in many tumours [37].

**Figure 6:**
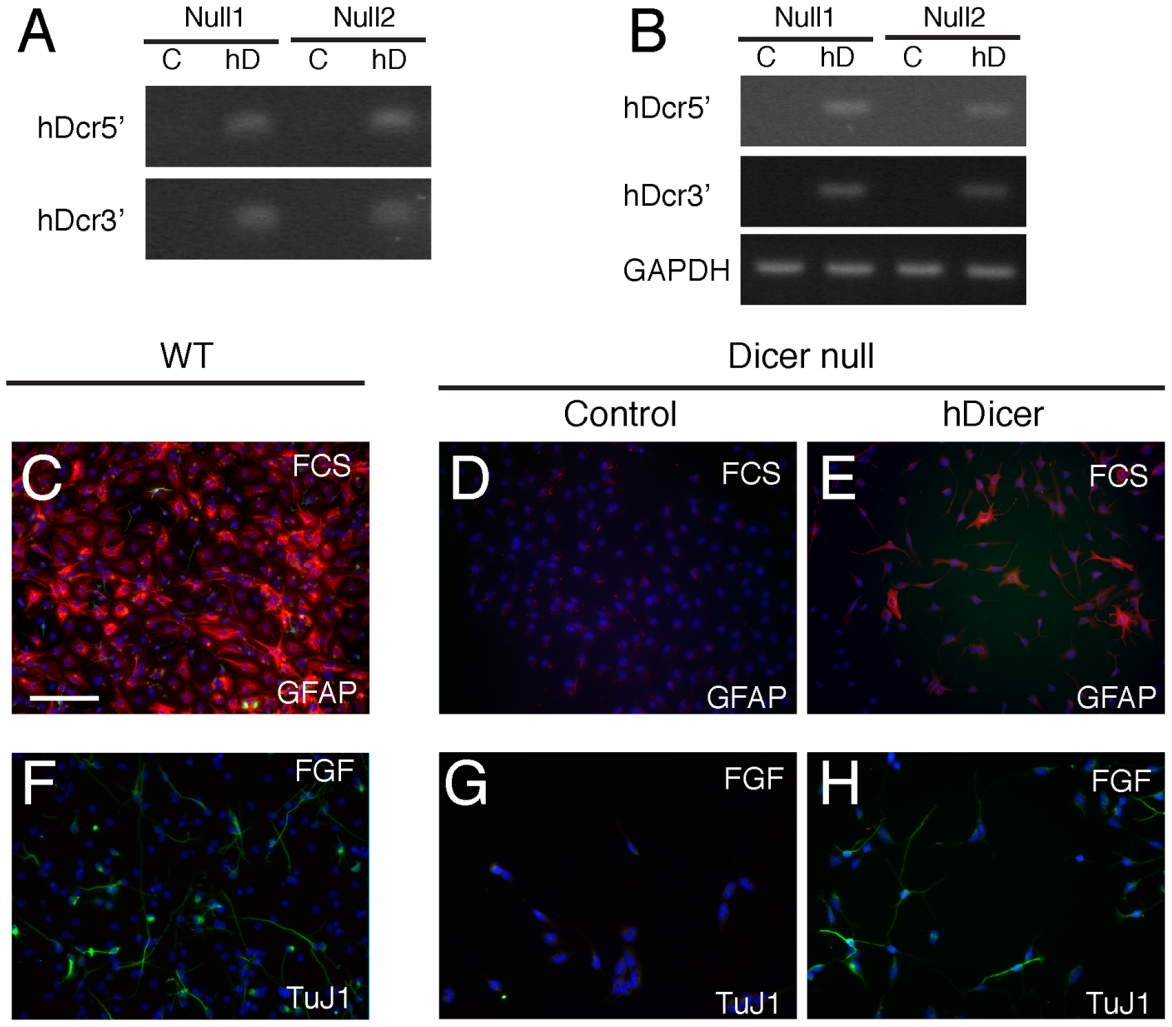
The differentiation block in Dicer-null NS cells can be reversed by reintroduction of Dicer. (A) Genomic PCR for human Dicer (hDicer) with two different sets of hDicer-specific primers in two lines of Dicer-null NS cells following infection with a luciferase-expressing virus (control, C) or a hDicer-expressing virus (hD) demonstrated the presence of hDicer DNA in the pFB-hDicer infected cells. (B) RT-PCR for hDicer RNA in virally-infected cells demonstrates expression of hDicer mRNA. (C–E) Wildtype control cells (C) differentiate to GFAP-expressing astrocytes 48 hours after exposure to FCS, in contrast with Dicer-null NS cells infected with a luciferase-expressing virus (D). Dicer-null NS cells infected with a hDicer-containing virus 24 hours before FCS addition recover the ability to generate GFAP-expressing astrocytes (E). (F–H) Similarly, wildtype cells (F) differentiate to Tuj1-expressing neurons upon withdrawal of EGF and maintenance of FGF2, in contrast with Dicer-null NS cells infected with a luciferase-expressing virus (G), the majority of which undergo apoptosis. Dicer-null NS cells infected with the hDicer-containing virus recover the ability to generate Tuj1-expressing neurons (H). Scale bar, 25 µm. Nuclei in all panels visualised with DAPI.

To test whether Dicer-null NS cells are irreversibly transformed to a proliferative phenotype incapable of differentiation, we acutely introduced human Dicer into the Dicer-null NS cells by retroviral misexpression (Fig. 6 A, B). Infected cells were then cultured under differentiating conditions 24 hours later. At this early stage of Dicer reintroduction, we found that the Dicer-null NS cells no longer undergo apoptosis upon EGF withdrawal. Importantly, they regained the ability to differentiate to both neurons and glia at high efficiency (Fig. 6C–H). We conclude that, despite the considerable phenotypic changes in the Dicer-null NS cells, acute reintroduction of Dicer corrected the major features of the phenotype, most notably their ability to differentiate. Therefore, Dicer-null NS cells are not transformed and have not irreversibly altered their neurogenic and gliogenic potential.

## Discussion

While many microRNAs are expressed in the developing nervous system in stem cells and differentiating neurons, the roles of microRNAs in key aspects of neural stem cell biology are still not well understood. MicroRNAs have been shown to positively and negatively regulate a variety of processes in the vertebrate nervous system, including morphogenesis of the nervous system in zebrafish [38], terminal differentiation of olfactory neurons [11] and the morphology of dendritic spines [39]. In several non-neural stem cell types and in germ cells, microRNAs have been found to be required for stem cell maintenance (for review, see [40]). Recently, it was found that loss of Dicer reduces the efficiency of neural stem cell derivation from the mouse cerebral cortex, and the self-renewal and differentiation of those cells [20]. Dicer has also now been shown to be required for the developmental change in the competence of retinal progenitor cells [18]. We find here that NS cells self-renew normally in the absence of Dicer but fail to differentiate due to marked phenotypic changes that block their ability to fully execute a differentiation programme. Importantly, although Dicer-null cells are highly abnormal in many respects, their ability to differentiate is restored shortly after the reintroduction of Dicer.

Low amounts of microRNAs appear to be sufficient for the microRNA system to function appropriately: we and others have found that the genesis of differentiated cell types occurs in many systems in the presence of low amounts of microRNAs [15], [16], [41], [42]. To address whether microRNAs are absolutely required for neural stem cell self-renewal and neurogenesis, we generated neocortical neural stem cell lines lacking Dicer that also lacked all microRNAs. In contrast to a recent report [20], NS cell lines were derived from the E12 mouse cortex at equal efficiency as from control cortices. We attribute this to the difference in the time of Dicer deletion in the two studies and the subsequent perdurance of microRNAs: we found that deletion of Dicer with Nestin-Cre results in microRNAs being present at approximately 40% of wild-type levels at E15. Therefore, the derivation of NS lines from the E12 Dicer null cortex begins when microRNAs are still present, and those lower levels of microRNAs are sufficient for NS cell derivation.

Having established Dicer-null NS cell lines, we cultured those cells for several weeks to allow the persisting microRNAs to be turned over, confirming the absence of all microRNAs assayed by microarray profiling. In that system, neural stem cell self-renewal was unaffected by the absence of Dicer and microRNAs, and these cell lines could be maintained indefinitely. However, Dicer-null NS cells were incapable of either gliogenesis or neurogenesis, and were unusually sensitive to EGF withdrawal-induced apoptosis. Regardless of the process used to promote the production of differentiated progeny, whether by growth factor withdrawal or by the use of specific (BMP4) or broad (foetal calf serum) inductive signals, Dicer-null NS cells effectively do not generate differentiated progeny.

Neurogenesis and neuronal differentiation continue in the cerebral cortex and retina following Dicer deletion [15], [16], [17], [18]. However, in both parts of the CNS there is a marked increase in apoptosis in the progenitor cell population [15], [16], [18]. The data presented here provide some clarification for the basis of that apoptosis: neural stem and progenitor cells lacking Dicer and microRNAs can self-renew, but undergo apoptosis when they attempt neurogenesis. We cannot ascertain exactly when this apoptosis occurs, whether it is during the later stages of the cell cycle or upon exiting cell cycle and initiating a neuronal differentiation program. With respect to glial differentiation *in vitro*, Dicer-null NS cells do not undergo apoptosis under those conditions, but do fail to terminally differentiate.

Although Dicer-null NS cells are grossly similar to their wild-type counterparts, expressing Nestin and Sox2, for example, loss of Dicer and microRNAs results in considerable changes in mRNA transcript levels in NS cells, with sets of both up- and down-regulated transcripts. These changes in mRNA levels would be predicted to have substantial effects on NS cell self-renewal and differentiation. The overall gene expression phenotype of Dicer-null NS cells is complex and cannot simply be defined as corresponding to a more or less well differentiated neural stem cell type: transcripts found in neurons and glia are at comparatively high levels in Dicer-null NS cells, along with many positive cell cycle regulators and genes that drive self-renewal or repress differentiation. Functionally, however, the dominant phenotype observed *in vitro* is an inability to generate differentiated progeny, with self-renewal effectively uncompromised in culture.

Notable among the transcripts upregulated in the Dicer-null NS cells is a number of genes normally associated with stem cell maintenance, including Prominin-1 (CD133) and Hmga2. The transcriptional regulator Hmga2 is normally expressed in neural stem cells early in embryonic development, where it actively drives self-renewal by indirectly repressing expression of negative regulators of cell cycle progression [43]. Previous studies have shown that the reduction in Hmga2 during development is mediated by let-7 family microRNAs [43], microRNAs expressed in wild-type NS cells and absent from Dicer-null NS cells. Increased Hmga2 expression or Hmga2 gene amplification initiate pituitary tumour development, and increased Hmga2 expression is found in several tumour types [37]. Therefore, the high levels of Hmga2 found in Dicer-null NS cells would be predicted to bias NS cells to self-renewal and inhibit differentiation, and could potentially reflect a transformation of Dicer-null NS cells.

A common feature of many tumour types is a global reduction in microRNAs[44], [45]. Together with the increase in Hmga2 and the inability to differentiate, this raises the question as to whether Dicer-null NS cells are transformed in some manner, or bear similarities with glioma cells. Dicer-null NS cells do not appear to be transformed to an oncogenic phenotype, as they show a marked dependence on growth factor signalling for survival, whereas a classic hallmark of the cancer cell is its growth factor independence [46]. In addition, Dicer-null NS cells express many genes that are normally expressed in differentiating neurons, and not in neural stem cells, such as Doublecortin [47], and the transcription factors Foxp1 and Foxp2 [34]. Furthermore, Dicer-null NS cells are incapable of generating differentiated progeny, whereas glioma cells typically can differentiate to neurons or glia *in vitro* [48]. Finally, and most importantly, the inability of Dicer-null cells to differentiate is reversed by the acute reintroduction of Dicer. Therefore, we conclude that while highly abnormal in their gene expression, with subtle changes in their heterochromatin status and cell cycle kinetics, these cells have not irreversibly altered their phenotype. Interestingly, recent genetic studies in mice and humans have found that Dicer haploinsufficiency increases tumour incidence, but that both alleles of Dicer are rarely deleted in human tumour cells *in vivo* [49], again suggesting that somatic cells lacking all Dicer function do not become transformed.

The lack of dependence of NS cell self-renewal on Dicer and microRNAs, combined with the inability of those cells to generate differentiated progeny, has obvious parallels with the functions of Dicer and microRNAs in embryonic stem cells. Dicer or DGCR8 null ES cells can self-renew but fail to differentiate [13], [14]. DGCR8 null ES cells maintain inappropriate expression of stem cell regulators under differentiation-promoting culture conditions [14]. The persistent expression of stem cell regulators under differentiating conditions essentially renders DGCR8 null ES cells resistant to differentiation and this phenotype was attributed to a failure of the microRNA system to reduce the levels of those proteins during differentiation [14].

Data presented here suggest that an additional microRNA function in neural stem cells may be to modulate the levels of regulators of self-renewal to a level compatible with differentiation, effectively lowering the barrier to differentiation given the appropriate inductive signals. Dicer-null NS cells do respond to differentiation-inducing signals, up-regulating and down-regulating sets of transcripts in the same manner as wild-type cells. However, Dicer-null NS cells fail to complete a differentiation program, and in the case of gliogenesis this appears to be the result of not up-regulating expression of a large set of cell-specific transcripts characteristic of the differentiated astrocyte.

In conclusion, we have found that the dominant phenotype observed upon removal of all microRNAs in NS cells is a failure to maintain cellular homeostasis and to regulate critical transitions during differentiation. In support of this, we found that neural stem cells could proceed through the cell cycle and self-renew in the absence of Dicer and all microRNAs, were substantially different phenotypically from their wild-type counterparts and could not generate differentiated progeny. However, their ability to differentiate is restored by the reintroduction of Dicer, indicating that Dicer-null NS cells are not irreversibly transformed.

## Supporting Information

Table S1 microRNAs detected reproducibly in cortical NS cells in vitro.
(0.01 MB XLS)

Table S2 Limma analysis to identify genes with increased or decreased expression in Dicer-null NS cells compared with wild-type controls.
(1.01 MB XLS)

Table S3 Gene Ontology analysis of genes with increased or decreased expression in Dicer-null NS cells compared with wild-type controls.
(0.31 MB XLS)

Table S4 Contents of clusters of genes showing differential expression between BMP4-treated Dicer-null and wild-type NS cells.
(0.72 MB XLS)

Table S5 Expression of astrocyte-specific genes in BMP4-treated wild-type and Dicer-null NS cells.
(0.03 MB XLS)
